# Experimental Infection and Natural Contact Exposure of Dogs with Avian Influenza Virus (H5N1)

**DOI:** 10.3201/eid1402.070864

**Published:** 2008-02

**Authors:** Matthias Giese, Timm C. Harder, Jens P. Teifke, Robert Klopfleisch, Angele Breithaupt, Thomas C. Mettenleiter, Thomas W. Vahlenkamp

**Affiliations:** * Friedrich-Loeffler-Institut, Greifswald-Insel Riems, Germany

**Keywords:** highly pathogenic avian influenza (H5N1) virus, dog, cat, canine, feline, susceptibility, dispatch

## Abstract

Experiments that exposed influenza virus (H5N1)–infected cats to susceptible dogs did not result in intraspecies or interspecies transmission. Infected dogs showed increased body temperatures, viral RNA in pharyngeal swabs, and seroconversion but not fatal disease.

Highly pathogenic avian influenza (HPAI) virus (H5N1) has spread across Asia, Europe, and Africa. Transmission of the virus to felids has been repeatedly reported (*1**–**4*). Investigations also indicate virus transmission to dogs. A fatal infection was documented in Thailand (*5**,**6*). In central Thailand, seroprevalence of ≈25% among 629 village dogs was reported (*7*). The virus was also detected in 2 dogs on Bali (*8*). The often close contact between dogs and humans raises questions about the zoonotic potential and the role of dogs in transmission and adaptation of influenza virus (H5N1) to mammals.

## The Study

Experiments were performed on 2 groups of animals housed in different rooms in the high-containment animal facility (Biosafety Level 3+) at the Friedrich-Loeffler-Institut using the highly pathogenic influenza virus (H5N1) strain A/cat/Germany/R606/2006 (*2**,**9*). All experiments were approved by the ethics committee. The first group comprised 5 dogs and 3 cats; 3 cats and 3 dogs were in the second group. The dogs (beagles, 10–12 weeks of age) were obtained from Harlan Laboratories (Borchen, Germany). The cats (8–10 months of age) were obtained from Charles River Laboratories (Dublin, Ireland). All animals were seronegative to influenza by ELISA (Pourquier Blocking AI type A (Rhone Mtrieux, France) and negative to influenza H5 antigen in hemagglutination-inhibition assays (HI).

In the first group of animals, 4 dogs were inoculated oculo-nasopharyngeally with 10^6^ 50% egg infectious dose (EID_50_). From day 1 postinfection (p.i.) onwards, 1 uninfected dog and 3 uninfected cats were housed in the same containment room. The cats had the possibility of withdrawing and hiding, but they could also have direct contact with the dogs through 1 part of the cage fence. During the study the cats frequently had direct nose-to-nose contact with the dogs. For a realistic contact exposure setting, the cats were fed by using the dogs’ food and water bowls without prior cleaning. In the second group, 3 cats were inoculated oculo-nasopharyngeally with 10^6^ EID_50_. Three uninfected dogs were housed in the same containment room. Direct contact between the animals was similarly enabled as for group 1, and contact again occurred frequently. Two cats and 2 dogs housed in a separate room served as negative controls. Animals were monitored by physical examination and for viral excretion for 21 days by using pharyngeal and rectal swabs.

Conjunctivitis and elevated body temperatures (39.2°C to 39.7°C) developed within 2 days p.i. in all inoculated dogs (group 1). On day 4 p.i., the conjunctivitis had resolved and only 2 dogs had body temperatures >39°C. By day 6 p.i., body temperatures of all animals had declined to <39°C. No additional clinical signs were observed.

Viral RNA was detected in pharyngeal and rectal swabs by real-time reverse transcriptase–PCR (RT-PCR), according to the method of Spackman et al. (*10*). Infectious virus was detected by titration of swab fluid in MDCK cells. Among the pharyngeal and rectal swabs taken at days 0, 2, 4, 6, and 18 p.i., only the pharyngeal swabs taken on day 2 p.i. from 3 of the inoculated dogs were positive by RT-PCR. No infectious virus was isolated. Plasma and peripheral blood mononuclear cell (PBMC) samples taken on day 4 p.i. were negative for viral RNA.

One negative control dog and 2 inoculated dogs were euthanized on day 10 p.i. Sera derived from these animals were negative in the ELISA and HI tests. The serum of 1 dog euthanized on day 21 p.i., however, was positive in ELISA and in HI testing with a titer of 16. Pharyngeal swabs from this dog were also positive by RT-PCR (Table). At necropsy, no gross lesions were present that could be attributed to the influenza infection. Histopathologic examination of the liver showed a scant lymphocytic periportal infiltration in dog no. 3. Influenza virus nucleoprotein could not be detected by immunohistochemical tests in trachea, lungs, liver, kidney, adrenals, thyroid, spleen, lymph nodes, or thymus. Antibodies did not develop in the second dog, euthanized on day 21 p.i. This animal also never tested positive for viral RNA in swab samples by RT-PCR.

**Table:** Course of experimental influenza virus (H5N1) infection of dogs and in-contact animals*

Dog no.	EID_50_	Body temperature (dpi)					Pharyngeal swab (dpi)				Euthanized (dpi)	Antibodies	
		0	2	4	6		0	2	4	6		HI	ELISA
1	10^6^	38.5	39.2	38.8	38.8		–	+	–	–	10	–	(+)†
2	10^6^	38.4	39.7	39.1	38.8		–	+	–	–	10	–	(+)‡
3	10^6^	38.5	39.3	38.6	38.4		–	+	–	–	21	16	+
4	10^6^	38.7	39.3	39.1	38.6		–	–	–	–	21	–	–
5 (dog contact)	0	38.3	38.4	38.0	38.7		–	–	–	–	21	–	–
6 (cat contact)	0	38.4	38.2	38.4	38.5		–	–	–	–	21	–	–
7 (cat contact)	0	38.3	38.5	38.6	38.8		–	–	–	–	21	–	–
8 (cat contact)	0	38.4	38.5	38.2	38.3		–	–	–	–	21	–	–
9 (neg. control)	0	38.4	38.2	38.7	37.8		–	–	–	–	21	–	–
10 (neg. control)	0	38.3	38.6	38.8	38.0		–	–	–	–	21	–	–

*Elevated body temperatures (above 39°C) are underlined. Real time reverse transcription–PCR results are given as – (cycle threshold [ct] values >38) and + (ct values 31–38). Animal serum samples were examined by hemagglutination-inhibition (HI) using highly pathogenic influenza virus (H5N1) strain A/cat/Germany/R606/2006 (*9*) as antigen. Results are given as reciprocal titers. Competitive nucleoprotein ELISA results are given as – (inhibition <65%) and + (inhibition >65); EID_50_, 50% egg infectious dose; dpi, days postinfection. †Serum from dog no. 1 on dpi 10 showed 39.4% inhibition. ‡Serum from dog no. 2 showed 21.6% inhibition.

Sera of the inoculated dogs were investigated biochemically for all enzymes that could be analyzed with a FUJI DRI-CHEM 3500 i (Sysmex, Leipzig, Germany). Elevated liver enzymes have been reported in cats that were naturally infected with influenza virus (H5N1) (*2*). Two dogs showed increased aspartate aminotransferase (AST) values up to 110 U/L (reference values 33–52 U/L) on day 4 p.i. These dogs also showed elevated body temperature on days 2 and 4 p.i., and viral RNA was detected in pharyngeal swabs on day 2 p.i. Dog no. 2 (Table) also showed elevated creatine phosphokinase (CPK) values up to 441 U/L (reference values 54–361 U/L) on day 4 p.i. The cause of the elevated AST and CPK levels noted on day 4 p.i. could have been nonspecific muscle injury. This dog was euthanized on day 10; no muscle injury was observed at necropsy.

The uninoculated dog housed with the infected dogs, as well as the 3 uninfected contact cats in group 1, never showed clinical symptoms. None of the pharyngeal and rectal swab samples, PBMC, or sera derived from these contact animals was positive by RT-PCR. No specific antibodies were detected. Clearly, the virus was not transmitted to the contact dog and cats.

In the second group of animals, severe symptoms—including high body temperatures (>40°C), decreased activity, conjunctivitis, and labored breathing within 2 days p.i. similar to recent reports about influenza virus (H5N1) infections of cats (*11**,**12*)—developed in all infected cats. The cats excreted virus through the respiratory and digestive tract. Viral titers quantified in the pharyngeal swabs reached 50% tissue culture infectious dose 10^5^ between days 2 and 4 p.i. Two animals were euthanized because of their symptoms within 5 days p.i. The third infected cat recovered and had HI titers of 64 within 2 weeks p.i. No clinical symptoms developed in the 3 contact dogs, and pharyngeal swabs, PBMC, and sera were negative by RT-PCR. Specific antibodies did not develop in the dogs (Table).

## Conclusions

Dogs are susceptible to HPAI virus (H5N1) infection. In our study, they reacted with a transient rise in body temperature and in some instances with specific antibodies. Viral RNA was detected in pharyngeal swabs. Infectious virus could not be reisolated, and transmission of virus to a contact dog and cats did not occur. Contact exposure experiments of influenza virus (H5N1)–infected cats with uninfected dogs did not result in interspecies transmission. The different outcome of infection with the same dose of influenza virus (H5N1) suggests that cats are more susceptible than dogs to disease. However, the experiments were performed with healthy animals; concurrent infections, impaired immune functions, and changing viral characteristics might lead to aggravated infections. Also, since some dog breeds are genetically predisposed for certain viral and bacterial diseases, other breeds might be more susceptible to influenza virus (H5N1) infection (e.g., equine influenza virus [H3N8] caused disease predominantly in small groups of dogs of particular breeds, including greyhounds [*13*]). Therefore, dogs may have a role in adaptation of HPAI virus (H5N1) to mammals and its subsequent transmission to humans.
